# The influence of insulin resistance on cerebrospinal fluid and plasma biomarkers of Alzheimer’s pathology

**DOI:** 10.1186/s13195-017-0258-6

**Published:** 2017-04-26

**Authors:** Sarah Westwood, Benjamine Liu, Alison L. Baird, Sneha Anand, Alejo J. Nevado-Holgado, Danielle Newby, Maria Pikkarainen, Merja Hallikainen, Johanna Kuusisto, Johannes R. Streffer, Gerald Novak, Kaj Blennow, Ulf Andreasson, Henrik Zetterberg, Ulf Smith, Markku Laakso, Hilkka Soininen, Simon Lovestone

**Affiliations:** 1 Department of Psychiatry, Warneford Hospital, University of Oxford, Oxford, OX3 7JX UK; 2 Institute of Clinical Medicine, Neurology, University of Eastern Finland, 70211 Kuopio, Finland; 3 Institute of Clinical Medicine, Internal Medicine, University of Eastern Finland and Kuopio University Hospital, 70211 Kuopio, Finland; 4 Janssen Research and Development, Janssen Pharmaceutics NV, Turnhoutseweg 30, 2340 Beerse, Belgium; 5 Janssen Pharmaceutical Research and Development, 1125 Trenton-Harbourton Road, Titusville, NJ 08560 USA; 6 Institute of Neuroscience and Physiology, Department of Psychiatry and Neurochemistry, Sahlgrenska Academy at the University of Gothenburg, SE-431 80 Mölndal, Sweden; 7 Clinical Neurochemistry Laboratory, Sahlgrenska University Hospital, 431 80 Mölndal, Sweden; 8 Department of Molecular Neuroscience, UCL Institute of Neurology, Queen Square, London, WC1N 3BG UK; 9 Lundberg Laboratory for Diabetes Research, Department of Molecular and Clinical Medicine, Sahlgrenska Academy at the University of Gothenburg, 405 30 Gothenburg, Sweden; 10 Neurocenter, Neurology, Kuopio University Hospital, 70211 Kuopio, Finland

**Keywords:** Alzheimer’s disease, Plasma biomarkers, Cerebrospinal fluid biomarkers, Proteomics, Diabetes mellitus, Insulin resistance

## Abstract

**Background:**

Insulin resistance (IR) has previously been associated with an increased risk of developing Alzheimer’s disease (AD), although the relationship between IR and AD is not yet clear. Here, we examined the influence of IR on AD using plasma and cerebrospinal fluid (CSF) biomarkers related to IR and AD in cognitively healthy men. We also aimed to characterise the shared protein signatures between IR and AD.

**Methods:**

Fifty-eight cognitively healthy men, 28 IR and 30 non-IR (age and *APOE* ε4 matched), were drawn from the Metabolic Syndrome in Men study in Kuopio, Finland. CSF AD biomarkers (amyloid β-peptide (Aβ), total tau and tau phosphorylated at the Thr181 epitope) were examined with respect to IR. Targeted proteomics using ELISA and Luminex xMAP assays were performed to assess the influence of IR on previously identified CSF and plasma protein biomarker candidates of AD pathology. Furthermore, CSF and plasma SOMAscan was performed to discover proteins that associate with IR and CSF AD biomarkers.

**Results:**

CSF AD biomarkers did not differ between IR and non-IR groups, although plasma insulin correlated with CSF Aβ/tau across the whole cohort. In total, 200 CSF and 487 plasma proteins were differentially expressed between IR and non-IR subjects, and significantly enriched pathways, many of which have been previously implicated in AD, were identified. CSF and plasma proteins significantly associated with CSF AD biomarkers were also discovered, and those sensitive to both IR and AD were identified.

**Conclusions:**

These data indicate that IR is not directly related to the level of CSF AD pathology in cognitively healthy men. Proteins that associated with both AD and IR are potential markers indicative of shared pathology.

**Electronic supplementary material:**

The online version of this article (doi:10.1186/s13195-017-0258-6) contains supplementary material, which is available to authorized users.

## Background

Research results suggest that two global epidemics, Alzheimer’s disease (AD) and diabetes mellitus (DM), are connected pathophysiologically. Impaired glucose tolerance, hyperinsulinaemia and DM are associated with increased risk of dementia or AD [1–8], and AD patients have been reported to have reduced insulin sensitivity [9], with insulin concentrations often found to be elevated in plasma and decreased in cerebrospinal fluid (CSF) [10, 11]. The IR–AD association may arise due to a shared aetiology resulting in the presence of one mutually increasing the risk of the other, or IR may mechanistically lead to AD. Either way, IR is potentially a modifiable risk factor for AD; however, it is still unclear exactly how and at what stage IR and AD interact. Further, IR is strongly associated with atherosclerosis and vascular disease, and whether IR is directly associated with AD pathophysiology (amyloid plaques and tau pathology) or whether it fosters other types of pathology (e.g. cerebrovascular changes) that may cause cognitive impairment alone or together with AD pathology is an open question.

Cognitively normal individuals with IR are of great interest in our effort to gain an understanding of the antecedents of this problem. In cognitively healthy subjects, IR has been linked with increased loss of temporal grey matter and cognitive decline [12–14], hypometabolism in AD-related brain regions such as the hippocampus [15] and higher levels of CSF AD biomarkers [16]. These results suggest that IR is already contributing to AD pathology in the preclinical disease stage.

The current study investigated whether IR and other markers associated with DM (plasma glucose, CSF and plasma insulin) may act as an early endophenotype of AD pathology by examining levels of the best validated molecular biomarkers of AD, CSF levels of amyloid β (Aβ), total tau (T-tau) and tau phosphorylated at the Thr181 epitope (P-tau), in cognitively healthy, age and *APOE* ε4 genotype-matched IR and non-IR subjects. In addition we utilised targeted protein studies and untargeted proteomics to explore other potential biomarker associations with IR and their links with AD. For targeted studies we measured proteins previously associated with AD or neurodegeneration; in CSF assaying, neurofilament light chain (NFL), monocyte chemotactic protein-1 (MCP-1) and YKL-40 also known as chitinase-3-like-1 [17]; and in plasma, ficolin-2 (FCN2), fibrinogen gamma chain (FGG), complement factor H-related 1 (CFHR1) and apolipoprotein A-I (ApoA1) [18–20] (Baird et al., unpublished observations). For untargeted, exploratory proteomics we utilised a high-dimensionality aptamer capture array (SOMAscan; SomaLogic Inc., Boulder, CO, USA) to identify candidate CSF and plasma protein biomarkers related to IR and AD, and examine their concordance.

## Methods

### Subjects and clinical classification

Participants were selected from the Metabolic Syndrome in Men (METSIM) study performed at the University of Eastern Finland, Kuopio, Finland [21]. To be considered eligible, subjects had to have a normal glucose tolerance in an oral glucose tolerance test (OGTT) performed within the past 3 months. IR was defined as Matsuda insulin sensitivity index < 25th percentile in subjects with otherwise normal OGTT [22].

In total, 58 subjects (mean age = 62.66 years) were included in this study; 28 IR and 30 non-IR. The groups were matched for age and *APOE* haplotype. All subjects had normal cognition determined by living independently, no memory complaints and a Mini-Mental State Examination (MMSE [23]) score ≥ 25. In addition, subjects had no history of significant neurological disorders, no prior diagnosis of DM, no evidence of significant metabolic or endocrine disorder associated with risk of cognitive impairment and no family history of autosomal dominant, inherited AD.

### Blood collection and processing, and clinical assessments

All procedures were performed on a single visit to the Brain Research Unit at the University of Eastern Finland. The subjects arrived in the morning after an overnight fast. All subjects underwent a standardised clinical examination, including a review of recent medical history and concurrent medications, general physical examination (including diastolic and systolic blood pressure) and neurological examination. MMSE was performed by a qualified examiner. In addition, all subjects completed a Functional Activities Questionnaire (FAQ [24]).

Fasting blood samples were obtained from an antecubital vein after 12 hours of fasting for measurements of basic blood chemistry (electrolytes, creatinine, total protein and albumin), haematology (complete blood count), thyroid function (thyroxine and thyroid stimulating hormone) and metabolic function (e.g. fasting plasma glucose and insulin levels). Blood samples for glucose and insulin (collection on ice) analysis were centrifuged at +4 °C at 2400 × *g* for 10 minutes. Plasma was aliquoted and stored at –80 °C. Blood samples for proteomic analysis were drawn 2 hours after the subjects had eaten (mean = 2 hours 5 minutes, standard deviation = 0.006). All blood samples collected for proteomics were centrifuged within 30 minutes of venepuncture and plasma supernatant was collected, and thereupon sample aliquots were frozen at –80 °C until further use.

CSF samples were collected by lumbar puncture at the L3/L4 or L4/L5 interspace. All samples were obtained in the morning according to a standard protocol [25]. CSF samples for glucose analysis were taken on ice and analysed directly. CSF samples for insulin and biomarker analysis were taken on ice, gently mixed and centrifuged at +4 °C at 2400 × *g* for 10 minutes. Supernatants were aliquoted into polypropylene tubes, and stored at –80 °C until proteomic analysis.

### *APOE* genotyping

Genotyping was performed using the TaqMan Allelic Discrimination Assays (Applied Biosystems, USA). Participants were classified according to their *APOE* haplotype as *APOE* ε4-positive if they had one ε4 allele (ε2/4 or ε3/4) or two ε4 alleles (ε4/4), and as *APOE* ε4-negative if they had no ε4 alleles.

#### Basic blood chemistry and fasting glucose and insulin levels

Plasma glucose levels (mmol/l) were measured by enzymatic glucose hexokinase photometric assay (Konelab System reagents; Thermo Fischer Scientific, Vantaa, Finland). Plasma and CSF insulin concentrations (mU/l) were determined by a chemiluminometric immunoassay measurement (Liaison^®^ Insulin; DiaSorin S.p.A, Saluggia, Italy) and by a photometric ELISA (Ultrasensitive Insulin ELISA; Mercodia, Uppsala, Sweden).

#### CSF biomarkers of AD pathology

CSF concentrations of the 42 amino acid form of amyloid β (Aβ1–42), T-tau and P-tau were measured using sandwich ELISAs (INNOTEST; Fujirebio, Ghent, Belgium). These three markers reflect senile plaque pathology, neurodegeneration and tangle pathology, respectively [17]. CSF analyses were performed at the Clinical Neurochemistry Laboratory, Sahlgrenska University Hospital, Mölndal, Sweden, by board-certified laboratory technicians. All CSF samples were analysed in one batch, with paired samples from individual patients side by side on the same plate. Samples were randomised and the CSF analysis team was blind to the IR status of subjects.

#### Targeted proteomics

We determined levels of proteins in CSF and in blood previously associated with AD or neurodegeneration. In CSF we measured the MCP-1 concentration using a sandwich immunoassay with electrochemiluminescent detection (MSD Human MCP-1; Meso Scale Discovery, Gaithersburg, MD, USA). CSF concentrations of YKL-40 and NFL were measured using sandwich ELISAs (R&D Systems, Minneapolis, Minneapolis, USA, and NF-light ELISA kit, UmanDiagnostics AB, Umeå, Sweden, respectively). MCP-1 and YKL-40 are both markers of astroglial activation, whereas NFL is a marker of large-calibre axonal degeneration [17]. In plasma we chose to measure four previously identified plasma biomarker candidates of AD pathology using the same proteomic platform as previous discovery experiments [18, 19, 26] (Baird et al., unpublished observations). FCN2, FGG and CFHR1 proteins were measured by ELISA from Cusabio and USCN Life Science Inc. (catalogue numbers CSB-EL008551HU, SEC477Hu and CSB-EL005274HU). ApoA1 was measured by Luminex xMAP assay (catalogue number HNDG1MAG-36 k; Merck Millipore). Proteins were measured in duplicate for every sample, and the average value was taken forward for statistical analyses. All CSF analyses were performed at the Clinical Neurochemistry Laboratory, Sahlgrenska University Hospital, Mölndal, Sweden and plasma analyses at the University of Oxford and King’s College London, UK.

#### Untargeted exploratory proteomics

SOMAscan (SomaLogic Inc.) is an aptamer-based assay allowing for the simultaneous measurement and quantification of 3615 proteins by 4006 unique SOMAmers (Slow Off-rate Modified Aptamers). The assay uses chemically modified nucleotides to transform a protein signal into a nucleotide signal that can then be quantified using relative florescence on microarrays [27]. A single SOMAscan assay was performed for each plasma and CSF sample.

### Statistical analysis

Statistical analyses were performed using R (version 3.2.0), SPSS (version 21) and DAVID (version 6.7). The distribution of Matsuda ISI, plasma and CSF insulin, CSF T-tau, CSF P-Tau, CSF NFL and CSF MCP were non-normal and their values were logarithmically transformed (log_10_) for statistical analysis. Additionally, all CSF and plasma protein values measured in untargeted and targeted proteomic experiments were log_10_ transformed. Type 1 error was monitored by modelling combinational probabilities, as detailed in ‘Modelling combinatorial probabilities’.

Clinical characteristics of subjects with and without IR are presented as the mean ± SD for continuous variables, or as the count (percentage) for the categorical variable (*APOE* ε4 status). Inter-group differences were analysed using the Mann–Whitney *U* test.

Spearman’s rank correlation was calculated between both plasma and CSF insulin values and CSF markers of AD pathology.

#### Targeted proteomics

To determine whether each of the proteins was differentially expressed between IR and non-IR men, Mann–Whitney *U* tests associating the concentration of each protein with IR status were run. To identify how well the resulting significant protein(s) could explain the variance in IR group assignment, logistic regression models were run including the additional variables age and body mass index (BMI). The optimal model was assessed further using receiver operating characteristic (ROC) and area under the curve (AUC) statistics.

Spearman’s rank correlation was calculated between each protein and each of the AD biomarker measurements (e.g. CSF Aβ). Significant correlations were re-tested within each IR group independently, to determine any influence of IR status.

#### Untargeted exploratory proteomics

To determine whether each of the 3615 proteins were differentially expressed between IR and non-IR, regression models associating the concentration of each CSF and plasma protein with IR status were run, controlling for age.

Plasma proteins that were differentially expressed at a significance level of *p* < 0.05 were nominated for pathway analysis. We evaluated the biological significance of the enriched proteins using pathway analysis. We used the DAVID Bioinformatics Resource (version 6.7) Functional Annotation tool and performed enrichment analysis on the KEGG database. The differentially expressed plasma proteins (*p* < 0.05) were input as our ‘gene list’ and probabilities were assigned to the distribution of proteins observed in the differentially expressed list versus those expected under a random draw of *n* proteins from the total set of proteins, where *n* is the number of differentially expressed proteins.

Stability selection regression with LASSO was used to identify the optimal multivariate plasma protein signatures that differentiate between IR and non-IR. Furthermore, significant plasma proteins differentially expressed between IR and non-IR subjects were compared with previously reported SOMAscan results from an AD vs control study (*p* < 0.05) [20] to identify common proteins from the two analyses.

Spearman’s rank correlation was calculated between each CSF and plasma protein and each of the AD CSF biomarker measurements. VENNY (version 2.1, [28]) was utilised to compare lists of significant proteins and identify those significantly related to IR status as well as the three most validated CSF markers of AD pathology; CSF Aβ, T-tau and P-tau.

#### Modelling combinatorial probabilities

Given a combination of outcomes (e.g. IR status, Aβ, T-tau and P-tau, all of them for either CSF or plasma), the question remains what is the probability of finding the given number of proteins being associated with all these outcomes from chance alone.

If we assume that an outcome follows a binomial distribution, the probability of a protein being associated with the outcome is trivially equal to the selected *p* value ‘*p*’ [29]. Meanwhile, if we consider the outcomes to be statistically independent of each other, the probability of a given protein being associated with ‘*k*’ out of ‘*K*’ outcomes (and not associated with the other ‘*K* – *k*’ outcomes) is:

1 $$ p(k)={\left(1- p\right)}^{K- k}{p}^k\left(\begin{array}{c}\hfill k\hfill \\ {}\hfill K\hfill \end{array}\right). $$

With these probabilities we can further calculate the averages of interest. Given a single outcome (e.g. CSF T-tau), the average number of proteins that would be associated with it under *p* value ‘*p*’ would simply be ‘*N* × *p*’, *N* being the total number of proteins tested (e.g. for a single outcome such as CSF T-tau, this would be 3615 × 0.05 ≈ 181). Meanwhile, the average number of proteins that would be associated with *k* out of *K* outcomes when measuring *N* proteins (we denote this statistical variable as ‘*x*’) would be:

2 $$ E(x)= N{\left(1- p\right)}^{K- k}{p}^k\left(\begin{array}{c}\hfill k\hfill \\ {}\hfill K\hfill \end{array}\right). $$

## Results

### Clinical characteristics and inter-group differences

The clinical characteristics of the study groups are presented in Table 1, along with the Mann–Whitney inter-group difference significance level. Concentrations of AD-related CSF biomarkers did not differ between IR and non-IR subjects (*p* > 0.05) suggesting that, as a group, people with IR do not have a higher level of preclinical AD pathology.

**Table 1:** Clinical characteristics of study population and inter-group difference significance levels (Mann–Whitney)

	Insulin non-resistant (*n* = 30)		Insulin resistant (*n* = 28)		Significance (*p* value)
Age (years)	62 ± 5	(55–69)	63 ± 4	(55–70)	0.33
MMSE, total score	29 ± 1	(25–30)	29 ± 1	(25–30)	0.44
BMI (kg/m2)	28.9 ± 1.8	(27.1–35.2)	30.1 ± 2.3	(27.1–36.0)	0.02*
*APOE* genotype (count, % ε4+)	13	43.3%	10	35.7%	0.56
Matsuda	9.1 ± 3.1	(6.4–17.2)	2.1 ± 0.3	(1.5–2.5)	0.00*
P-glucose (mmol/l)	5.7 ± 0.4	(4.8–6.7)	6.0 ± 0.6	(5.1–7.4)	0.12
P-insulin (mU/l), LIAISON	7.7 ± 3.8	(1.5–19.5)	19.6 ± 8.1	(8.0–43.2)	0.00*
P-insulin (mU/l), ELISA	5.4 ± 2.5	(1.4–12.3)	13.9 ± 6.3	(5.9–31.6)	0.00*
CSF insulin (mU/l), ELISA	0.15 ± 0.12	(0.10–0.64)	0.22 ± 0.15	(0.1–0.7)	0.00*
Aβ-42 (pg/ml), Fujirebio	856 ± 195	(530–1256)	888 ± 204	(531–1313)	0.59
T-tau (pg/ml), Fujirebio	256 ± 122	(107–670)	283 ± 110	(144–694)	0.16
P-tau (pg/ml), Fujirebio	44 ± 18	(20–105)	52 ± 28	(25–183)	0.10
CSF Aβ/tau	3.75 ± 1.0	(0.87-6.12)	3.38 ± 0.9	(1.31-5.04)	0.13

Data presented as mean ± SD (minimum–maximum) or as count (percentage) for the categorical variable (*APOE* ε4 status)

*MMSE* Mini-Mental State Examination, *BMI* body mass index, *APOE* apolipoprotein E, *ELISA* enzyme-linked immunosorbent assay, *CSF* cerebrospinal fluid, *Aβ* amyloid beta, *T-tau* total tau, *P-tau* tau phosphorylated at the Thr181 epitope

*Significant at *p* < 0.05

However, although the group analysis in this small study did not support the primary hypothesis, we noted a wide range of plasma insulin levels, indicative to some extent of the degree of IR and overlapping between the two groups. We therefore performed a continuous variable analysis between plasma insulin and markers of AD pathology using Spearman’s correlation across both IR and non-IR groups. This analysis showed a small but significant correlation between plasma insulin and CSF Aβ/tau ratio, the marker most indicative of AD pathology. This correlation was driven entirely by the association between plasma insulin and tau levels (Table 2).

**Table 2:** Spearman’s rank correlation results of plasma and CSF insulin with CSF markers of AD pathology

		CSF Aβ/tau	CSF Aβ	CSF T-tau	CSF P-tau
P-insulin (LIAISON)	Coefficient	−0.302	0.182	0.310	0.249
	Significance	0.021*	0.172	0.018*	0.060
P-insulin (ELISA)	Coefficient	−0.277	0.183	0.299	0.224
	Significance	0.035*	0.169	0.023*	0.091
CSF insulin (ELISA)	Coefficient	0.039	0.098	−0.002	−0.111
	Significance	0.771	0.466	0.990	0.406

*CSF* cerebrospinal fluid, *AD* Alzheimer’s disease, *Aβ* amyloid beta, *T-tau* total tau, *P-tau* tau phosphorylated at the Thr181 epitope, *ELISA* enzyme-linked immunosorbent assay

*Significant at *p* < 0.05

### Targeted proteomics results

We then measured three proteins in CSF that have previously been associated with AD pathology: NFL, MCP-1 and YKL-40, markers of axonal degeneration and astroglial activation respectively [17]. None of these markers differed in men with IR compared with those without (*p* > 0.05). We next measured four proteins in plasma previously associated with AD pathology; FCN2, FGG, APOA1 and CFHR1. Of these, only FCN2 was significantly differentially expressed between the IR and non-IR subjects (*p* = 0.014, *β* = –0.57). FCN2 was reduced in the IR group compared with non-IR.

**Fig. 1:**
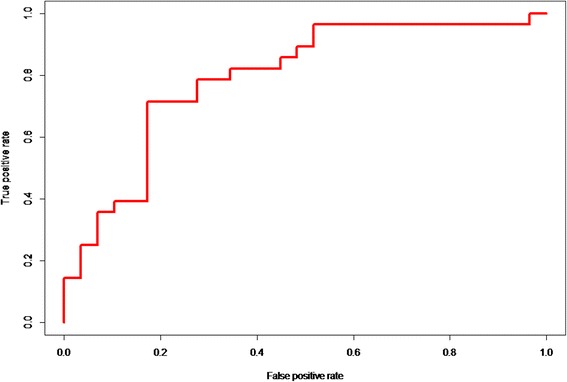
Classifier performance of the ‘FCN2 + BMI + Age model’ for IR group assignment. AUC = 0.79

Akaike information criterion comparison revealed that the best quality model for group classification was ‘FCN2 + BMI + Age’ (*p* = 0.007). This model was therefore selected for ROC and AUC statistical analyses. Figure 1 displays the ROC curve illustrating the classifier performance of the model (AUC = 0.79, sensitivity = 71%, specificity = 83%, accuracy = 77%).

### Protein association with AD pathology and determining the influence of IR

Because previous research had identified the targeted plasma proteins as candidate biomarkers of AD pathology, we next performed a correlation analyses between these proteins with CSF Aβ and with CSF T-tau and P-tau measures to determine whether their biomarker ability replicated in this cognitively healthy cohort. Spearman’s rank identified only one significant correlation: FCN2 significantly negatively correlated with CSF Aβ (*r*_s_ = –0.32, *p* = 0.014). To determine whether this correlation was influenced by IR status, Spearman’s rank correlation between FCN2 and CSF Aβ was performed within IR and non-IR subject groups independently. FCN2 was found to be significantly associated with CSF Aβ in the IR group (*r*_s_ = –0.656, *p* < 0.001), but not in the non-IR group (*p* = 0.71).

### Untargeted exploratory proteomics results

Finally we performed an exploratory proteomics study in plasma and in CSF using a high-dimensionality aptamer capture array measuring 3615 proteins (SomaLogic Inc.). A total of 200 proteins in CSF and 487 proteins in plasma were significantly differentially expressed between IR and non-IR subjects (*p* < 0.05). Full results are reported in Additional files 1 and 2.

Pathway analysis of the 487 plasma proteins that were differentially expressed between IR and non-IR subjects (*p* < 0.05) revealed seven significantly enriched pathways; complement and coagulation cascades (*p* = 8.43 × 10^–9^), cytokine–cytokine receptor interaction (*p* = 1.56 × 10^–5^), axon guidance (*p* = 0.006), type I DM (*p* = 0.006), the Jak-STAT signalling pathway (*p* = 0.010), apoptosis (*p* = 0.024) and the GnRH signalling pathway (*p* < 0.044).

Stability selection regression identified an optimal multivariate signature of 47 plasma proteins that could differentiate IR and non-IR, with AUC = 0.84, sensitivity = 77%, specificity = 75% and accuracy = 76% (Table 3).

**Table 3:** Plasma proteins included in the IR group classifier model and their rank order

Ranking	Plasma protein
1	Sialic acid-binding Ig-like lectin 9
2	MHC class I polypeptide-related sequence A
3	Macrophage metalloelastase
4	Teratocarcinoma-derived growth factor 1
5	Haemoglobin
6	Myosin-binding protein C, slow-type
7	Alkaline phosphatase, tissue-non-specific isozyme
8	Lipopolysaccharide-binding protein
9	Type II inositol 1,4,5-trisphosphate 5-phosphatase
10	WD repeat-containing protein 1
11	Immunoglobulin D
12	Tyrosine-protein phosphatase non-receptor type substrate 1
13	Collagen alpha-3(VI) chain
14	Chitotriosidase-1
15	Protein-glutamine gamma-glutamyltransferase E
16	Carboxypeptidase A4
17	Low-affinity immunoglobulin gamma Fc region receptor II-a
18	Paired immunoglobulin-like type 2 receptor alpha
19	CD177 antigen
20	Bone sialoprotein 2
21	Odorant-binding protein 2b
22	Epididymis-specific alpha-mannosidase
23	Protein S100-A13
24	Legumain
25	Multimerin-2
26	Amphoterin-induced protein 1
27	Glyceraldehyde-3-phosphate dehydrogenase
28	Complement C3b
29	Gastrokine-2
30	Periostin
31	C-X-C motif chemokine 11
32	Haptoglobin
33	Histone-lysine *N*-methyltransferase SETMAR
34	Urotensin-2 receptor
35	Leucine-rich repeat neuronal protein 1
36	Apolipoprotein L1
37	Collagen alpha-1(VIII) chain
38	Paired immunoglobulin-like type 2 receptor alpha
39	Thrombin
40	Stannin
41	Myeloid cell surface antigen CD33
42	Protein LEG1 homologue
43	Lactadherin
44	Histo-blood group ABO system transferase
45	Chitinase-3-like protein 1
46	Trefoil factor 1
47	C-reactive protein
48	Complement C1q subcomponent

*IR* insulin resistance

From the 487 proteins significantly differentially expressed between IR and non-IR subjects, 25 had previously been identified as significantly differentially expressed between AD and control subjects using the SOMAscan assay (*p* < 0.05, Table 4) [20].

**Table 4:** SOMAscan measured plasma proteins differentially expressed between both IR vs non-IR and AD vs control subjects

Protein name	Uniprot ID
40S ribosomal protein S3	P23396
Afamin	P43652
Alpha-(1,3)-fucosyltransferase 5	Q11128
Alpha-1-antichymotrypsin	P01011
Calcineurin	Q08209 P63098
cAMP-dependent protein kinase catalytic subunit alpha	P17612
cAMP-regulated phosphoprotein 19	P56211
CD209 antigen	Q9NNX6
Ciliary neurotrophic factor receptor subunit alpha	P26992
Coagulation Factor V	P12259
Fetuin-B	Q9UGM5
Fibronectin Fragment 4	P02751
Gelsolin	P06396
Growth hormone receptor	P10912
Insulin-like growth factor-binding protein 2	P18065
Kallikrein-8	O60259
Kininogen-1	P01042
Lysosomal protective protein	P10619
Mitogen-activated protein kinase 12	P53778
*N*-acetylglucosamine-6-sulfatase	P15586
Prolyl endopeptidase FAP	Q12884
Protein disulfide-isomerase A3	P30101
P-selectin	P16109
Retinoblastoma-associated protein	P06400
Wnt inhibitory factor 1	Q9Y5W5

*IR* insulin resistance, *AD* Alzheimer’s disease

**Fig. 2:**
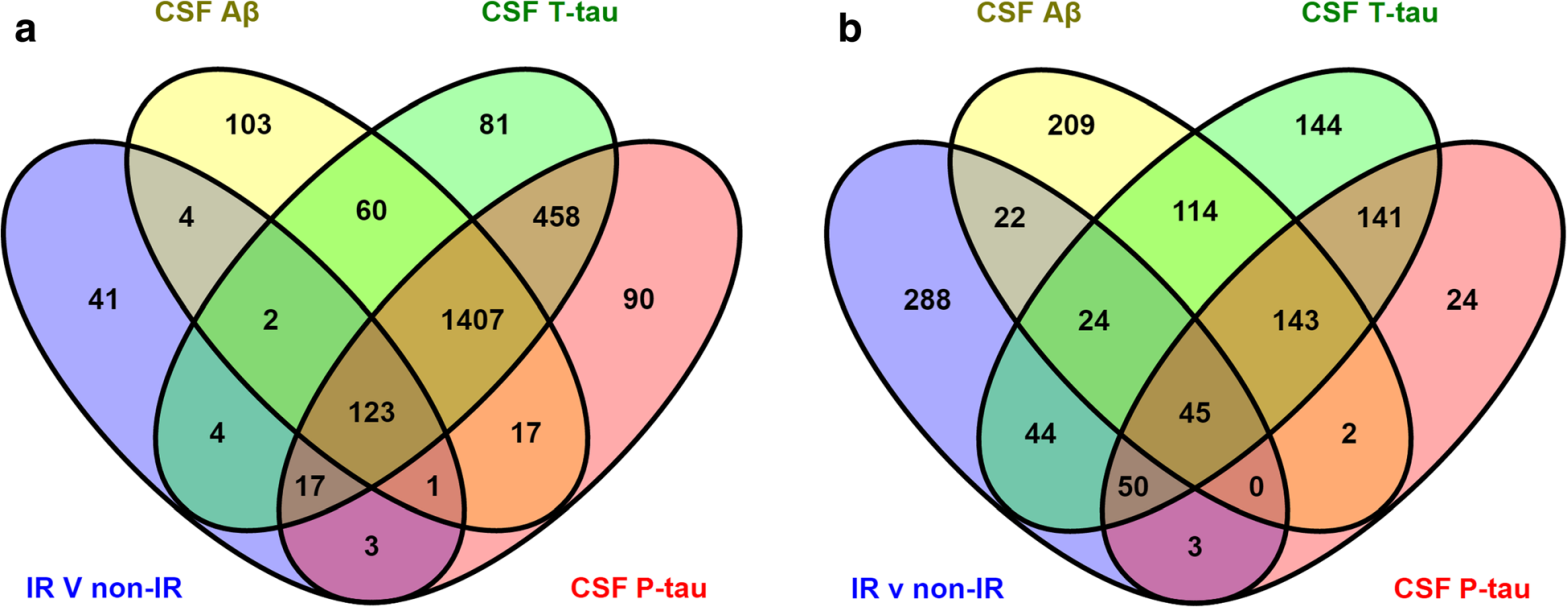
Venn diagrams displaying the number of significant CSF and plasma proteins for each statistical test. Number of CSF (**a**) and plasma (**b**) proteins significantly related to IR status (*blue*), CSF Aβ (*yellow*), CSF T-tau (*green*) and CSF P-tau (*red*) (Colour figure online). *CSF* cerebrospinal fluid, *Aβ* amyloid beta, *T-tau* total tau, *P-tau* tau phosphorylated at the Thr181 epitope, *IR* insulin resistance (Colour figure online)

Given that IR is a risk factor for AD, we hypothesised that proteins associated with IR would overlap with those associated with AD pathology. In order to test this hypothesis we first correlated all exploratory proteins with CSF Aβ and with CSF T-tau and P-tau measures. This correlation analysis found 2370 CSF and 965 plasma proteins significantly associated with one or more AD biomarker measures (*p* < 0.05, Additional files 3 and 4). We then performed list comparisons of proteins associated with these AD markers and with proteins associated with IR status using VENNY (version 2.1 [28]). We found 123 proteins in CSF and 45 proteins in plasma common to all three markers of AD pathology and shared with IR status (Fig. 2). Of these proteins, six were common to both plasma and CSF. These proteins, associated with both AD and IR in plasma and CSF, and therefore of most interest as potential markers indicative of shared pathology, are: Ciliary neurotrophic factor receptor subunit alpha; Discoidin, CUB and LCCL domain-containing protein 2; Ephrin-B2; Leucine-rich repeat-containing protein 4B; Neuronal growth regulator 1; and SLIT and NTRK-like protein 4.

Given a combination of outcomes (e.g. IR status, Aβ, T-tau and P-tau, all of them for either CSF or plasma), the question remains what is the probability of finding the given number of proteins being associated with all of these outcomes (e.g. 458 in the case of CSF T-tau and P-tau, see Fig. 2) from chance alone. The average number of proteins calculated to be expected by chance as significantly associated with zero, one, two, three and four out of four outcomes (i.e. ‘*K* = 4’) is, respectively, 2444, 514, 40.6, 1.43 and 0.0188. For the case of eight out of eight outcomes (i.e. ‘*k* = 8’ and ‘*K* = 8’, which corresponds to proteins being associated with all outcomes in both CSF and plasma), the average number would be 1.41 × 10^−7^. Therefore our finding that six proteins were consistently significantly associated with AD and IR in both CSF and plasma is not expected by chance alone.

## Discussion

This study examined the relationship between IR and AD pathology, through assessing the concentrations of IR and AD pathology biomarkers in the CSF and plasma of cognitively normal men with and without IR. To enable a clear assessment of IR influence on AD pathology, groups were closely matched for the two largest AD risk factors; age and *APOE* genotype. We found that concentrations of CSF markers of AD pathology did not significantly differ between IR and non-IR subjects, suggesting that, as a group, late middle-aged to aged men with IR are not more likely to be in the pre-clinical AD stage. These data are in line with previous findings utilising other approaches that suggest IR is not associated with increased amounts of AD pathology [30–32]. However, it is important to note that we do observe a trend towards increased CSF T-tau and P-tau in the IR group, and perhaps in a larger cohort this finding would have reached statistical significance.

However, to our knowledge only one prior study by Hoscheidt et al. in 2016 [16] has assessed the association of IR and CSF AD-related biomarkers in cognitively healthy middle-aged subjects. They demonstrated a minor positive association of IR with CSF soluble amyloid-β protein precursor β (sAPP-β) and Aβ42. Our results are therefore in disagreement with these findings. However there are key differences between the two study cohorts; Hoscheidt et al. [16] included only male and females with a parental family of history of AD, whereas our male-only study balanced *APOE* haplotypes across the groups to minimise the influence of this AD risk factor upon our results. Additionally IR measures differ between studies; Hoscheidt et al. [16] used the homeostatic model assessment of insulin resistance (HOMA-IR), reflecting mainly liver IR in the fasting state. In contrast, our study used the Matsuda ISI, which measures IR during glucose stimulation, and our previous study demonstrated that the Matsuda ISI has additional value for IR detection beyond the ability of HOMA-IR [33].

Although we did not find significant AD pathology differences between IR groups, a significant relationship was reported between continuous values of plasma insulin and CSF Aβ/tau driven by the correlation between insulin levels and levels of tau protein, suggesting a link between IR and neuronal degeneration. This finding is in line with preclinical studies from animal models also demonstrating an association between hyperinsulinaemia and tau pathology [34, 35].

Targeted CSF protein studies were in line with the studies of CSF biomarkers of AD pathology because none of the three markers previously associated with AD were significantly increased in IR. In blood we investigated the influence of IR on four previously identified plasma protein biomarker candidates of AD pathology: Ficolin-2 (FCN2; previously associated with brain atrophy [20] and CSF Tau/Aβ (Baird et al., unpublished observations)), fibrinogen gamma chain (FGG; previously associated with brain amyloid PET [18, 19]), complement factor H related 1 (CFHR1; previously associated with brain amyloid PET [18] and CSF Tau/Aβ (Baird et al., unpublished observations)) and apolipoprotein A-I (ApoA1; previously associated with brain amyloid PET [18, 19] and CSF Tau/Aβ (Baird et al., unpublished observations)). Of these proteins, only FCN2 was significant; with a reduction in FCN2 related to IR status. Furthermore, classification analyses showed that this single protein, plus BMI and age, performed well in predicting group assignment (AUC = 0.79). Based on previous IR research, this relationship was as expected because a reduction in ficolin-3, a protein structurally and functionally similar to FCN2, has previously been identified as a biomarker of type 2 diabetes [36]. Moreover, FCN2 was also found to be related to CSF AD pathology in our cohort; with a significant negative association found with CSF Aβ, driven by the IR group. This replicates previous AD research (Baird et al., unpublished observations) demonstrating a negative correlation between Aβ and FCN2 in a non-dementia cohort, and furthermore demonstrates an interaction effect of IR and AD on FCN2. Further investigation is required to clarify this relationship, but because FCN2 functions as a mediator of the lectin complement pathway our results may indicate that lectin complement disturbance, influenced by IR status, is a prerequisite for AD pathology.

We next reported 200 CSF and 487 plasma proteins significantly related to IR, and a group classification model of 47 plasma proteins which could predict IR status with an AUC of 84%. This analysis provides further insight into proteins affected by IR mechanisms, many of which have been identified previously, and allowed us to subsequently identify overlap with proteins/pathways known to be associated in AD. Pathway analysis of the differentially expressed proteins in plasma showed seven significantly enriched pathways, and many of these biological pathways have been implicated previously in AD [18, 37–39]. To determine whether the IR-related proteins had also been identified previously as AD-related proteins, our results were directly compared with those from Sattlecker et al. [20], a study which identified proteins differentially expressed between AD and healthy control subjects, using the same proteomic platform (SOMAscan). Sattlecker et al. used a smaller SOMAscan assay size, 1300 proteins compared with our 4000, but from the comparison of 1300 proteins an overlap of 25 significant proteins between the two analyses was still found. These 25 proteins are therefore sensitive to both IR and AD, and further investigation is needed to identify the common mechanisms involved.

Untargeted exploratory proteomics also identified many candidate biomarkers of AD pathology in this cohort. Many of these proteins replicate previously identified candidate AD biomarkers, but at an earlier pre-clinical disease stage. Using the VENNY list comparison tool we identified proteins which were significantly related to IR as well as three of the most validated CSF markers of AD pathology: Aβ, T-tau and P-tau. One of the most notable plasma proteins common to all four tests is clusterin, because previous research has identified clusterin as one of the most promising plasma protein biomarker candidates of AD pathology. Here, plasma clusterin was reduced with IR status, and also negatively associated with CSF Aβ4 2, T-tau and P-tau.

Six proteins were consistently significantly related to IR and AD pathology measures (Aβ, T-tau and P-tau) in both CSF and plasma: Ciliary neurotrophic factor receptor subunit alpha (CNTFR); Discoidin, CUB and LCCL domain-containing protein 2 (DCBLD2); Ephrin-B2 (ENFB2); Leucine-rich repeat-containing protein 4B (LRRC4B); Neuronal growth regulator 1 (NEGR1); and SLIT and NTRK-like protein 4 (SLITRK4). These proteins are involved in functions such as cell adhesion (ENFB2, NEGR1, LRRC4B), cell signalling (DCBLD2, ENFB2), neuronal survival (CNTFR), neurite/neuron growth (NEGR1, SLITRK4) and JAK-STAT signalling (LRRC4B, CNTFR). These pathways may therefore be mutually influenced by IR and AD.

The limitations of this study need to be acknowledged. Our study used a small and specific cohort: 58 middle-aged, older Finnish men. Without further testing we therefore cannot generalise these results to other demographics, and replication of the findings reported here is needed to determine their strength across cohorts. A cognitively healthy cohort with AD and IR measures is rare, however, and, although limited, our findings will help inform future investigations.

## Conclusions

Overall, the results of this study may be useful in the detection of cognitively healthy subjects who are at higher risk for AD. Although our results suggest that IR is not directly related to the level of AD pathology in cognitively healthy individuals, we do see an influence of IR on AD pathology biomarkers. Further research is required to fully understand this interaction, and additionally to investigate insulin levels independent of IR status. Additionally, we identified proteins that are associated with both AD and IR in both plasma and CSF, and are therefore potential markers indicative of shared pathology. These proteins also provide an insight into biological pathways mutually influenced by IR and AD.

## Additional files

Additional file 1: Table presenting CSF proteins differentially expressed between IR and non-IR subjects (*p* < 0.05). (CSV 12 kb)

Additional file 2: Table presenting plasma proteins differentially expressed between IR and non-IR subjects (p < 0.05) (CSV 25 kb)

Additional file 3: Table presenting correlation results of CSF SOMAscan proteins with CSF markers of AD pathology (CSV 436 kb)

Additional file 4: Table presenting correlation results of plasma SOMAscan proteins with CSF markers of AD pathology (CSV 442 kb)

## Abbreviations

Aβ: Amyloid beta
AD: Alzheimer’s disease
ApoA1: Apolipoprotein A-I
APOE: Apolipoprotein E
AUC: Area under the curve
BMI: Body mass index
CFHR1: Complement factor H-related 1
CNTFR: Ciliary neurotrophic factor receptor subunit alpha
CSF: Cerebrospinal fluid
DCBLD2: Discoidin, CUB and LCCL domain-containing protein 2
DM: Diabetes mellitus
ELISA: Enzyme-linked immunosorbent assay
ENFB2: Ephrin-B2
FAQ: Functional Activities Questionnaire
FCN2: Ficolin-2
FGG: Fibrinogen gamma chain
IR: Insulin resistance
LRRC4B: Leucine-rich repeat-containing protein 4B
MCP-1: Monocyte chemotactic protein-1
METSIM: Metabolic Syndrome in Men
MMSE: Mini-Mental State Examination
NEGR1: Neuronal growth regulator 1
NFL: Neurofilament light chain
OGTT: Oral glucose tolerance test
P-tau: Tau phosphorylated at the Thr181 epitope
ROC: Receiver operating characteristic
SLITRK + A24:A304: SLIT and NTRK-like protein 4
SOMAmers: Slow Off-rate Modified Aptamers
T-tau: Total tau
YKL-40: Chitinase-3-like-1
